# Trastuzumab Deruxtecan in Metastatic Urothelial Carcinoma with NGS-Detected ERBB2 Amplification: A Four-Patient Real-World Case Series

**DOI:** 10.3390/curroncol33080456

**Published:** 2026-07-30

**Authors:** Giuseppe Di Lorenzo, Sara Di Lorenzo, Antonio Verde, Oriana Strianese, Luigi Leo, Carlo Buonerba

**Affiliations:** 1 Oncology Unit, “Andrea Tortora” Hospital, ASL Salerno, 84016 Pagani, Italy; 2 School of Medicine, UniCamillus-Saint Camillus International University of Health Sciences, 00131 Rome, Italy; 3 Associazione ORA ETS, 84134 Salerno, Italy; 4 Oncology Unit, ASL NA3 Sud, 80059 Torre del Greco, Italy

**Keywords:** urothelial carcinoma, bladder cancer, ERBB2 amplification, HER2, trastuzumab deruxtecan, antibody–drug conjugate, next-generation sequencing, precision oncology

## Abstract

**Background:** Next-generation sequencing (NGS)-detected ERBB2 amplification occurs in a subset of urothelial carcinomas, but its role as a treatment-selection marker for trastuzumab deruxtecan (T-DXd) remains uncertain. **Methods:** We retrospectively reviewed four patients with metastatic urothelial carcinoma treated with T-DXd in routine practice from 2024. Treatment selection was based on NGS-detected ERBB2 amplification because HER2 immunohistochemistry and in situ hybridization were unavailable. **Results:** Four men aged 65–76 years received T-DXd: one in the second line and three in the fourth or fifth line. The best radiological responses, abstracted from contemporaneous radiology reports and oncology medical records, were complete response in one patient, partial response in one, and stable disease in two. Three patients had previously received enfortumab vedotin. Documented adverse events included fatigue, anemia, diarrhea, rash, and leukopenia. No interstitial lung disease or pneumonitis was documented in the available records. **Conclusions:** These observations are descriptive and hypothesis-generating. They do not establish the efficacy or safety of T-DXd or validate ERBB2 amplification as a predictive biomarker, but they support prospective evaluation of genomic ERBB2 amplification when standard HER2 testing is unavailable.

## Simple Summary

Trastuzumab deruxtecan is an antibody–drug conjugate designed to deliver a cytotoxic drug to tumors with HER2-related alterations. Prospective studies have shown activity in bladder cancer selected by HER2 protein expression, but less is known about using ERBB2 gene amplification detected by next-generation sequencing when standard HER2 tests are unavailable. We describe four men with metastatic urothelial carcinoma treated on this basis in routine practice. Medical records documented one complete response, one partial response, and two cases of stable disease. Three patients had previously received enfortumab vedotin. Because the series is small, retrospective, and has incomplete biomarker information, it cannot establish effectiveness, safety, or the predictive value of ERBB2 amplification. The cases instead highlight a real-world genomic treatment-selection approach that should be evaluated prospectively together with standardized HER2 protein and gene testing.

## 1. Introduction

The first-line treatment of locally advanced or metastatic urothelial carcinoma has changed substantially. Enfortumab vedotin plus pembrolizumab is now the preferred regimen for patients eligible for combination therapy [1,2]. When this combination is contraindicated, unsuitable, declined, or unavailable, platinum-based chemotherapy remains an appropriate first-line option; in patients without progression after four to six cycles of platinum-based chemotherapy, switch-maintenance avelumab remains an evidence-based strategy [1,3]. The optimal sequence after first-line enfortumab vedotin plus pembrolizumab remains incompletely defined.

HER2, encoded by ERBB2, has long been investigated as a therapeutic target in urothelial carcinoma. Reported frequencies of ERBB2 amplification vary according to disease setting, specimen type, assay, and amplification threshold. A recent standardized analysis of advanced urothelial carcinoma specimens identified ERBB2 amplification in 11.6% of cases, while systematic reviews have emphasized the heterogeneity of HER2 testing and the imperfect correspondence between genomic amplification and protein expression [4,5]. ERBB2 amplification, HER2 protein expression, and activating ERBB2 mutations should therefore be regarded as related but non-interchangeable biomarker categories.

Trastuzumab deruxtecan (T-DXd) is a HER2-directed antibody–drug conjugate composed of a trastuzumab-based antibody, a cleavable linker, and a topoisomerase I inhibitor payload. DESTINY-PanTumor02 demonstrated activity across HER2-expressing solid tumors [6]. In the final bladder cancer analysis, 41 patients selected by HER2 immunohistochemistry received T-DXd, and 17 achieved an investigator-assessed objective response [7]. Additional tumor-agnostic studies have evaluated T-DXd in tumors with NGS-detected ERBB2 amplification or selected activating ERBB2 mutations [8,9].

The present report does not seek to provide a new efficacy estimate beyond these prospective studies. Rather, it describes four patients in whom T-DXd was selected on the basis of NGS-detected ERBB2 amplification because HER2 immunohistochemistry and in situ hybridization were unavailable, including three patients previously exposed to enfortumab vedotin. We report their individual treatment sequences, chart-documented radiological outcomes, adverse events, and the limitations of genomic-only biomarker selection.

## 2. Methods

This retrospective, single-center case series included four patients with histologically confirmed metastatic urothelial carcinoma of the bladder who received trastuzumab deruxtecan (T-DXd) at the Oncology Unit of the “Andrea Tortora” Hospital, ASL Salerno, Pagani, Italy. The patients initiated T-DXd during 2024, and the available medical records were reviewed through 21 July 2026. This record-review cutoff does not imply that every patient remained under active clinical follow-up through that date. T-DXd was administered off-label at 5.4 mg/kg intravenously every three weeks as individualized treatment after prior systemic therapy and was authorized according to local institutional procedures.

Molecular profiling was performed during routine clinical care. For Patients 3 and 4, the available records identified archival bladder tumor material as the tested specimen, although the exact sampling dates could not be retrieved. For Patient 3, the available report documented an estimated ERBB2 copy number of 10.4 and a pathogenic ERBB2 p.Asp277Tyr (c.829G>T) variant with a variant allele frequency of 81.9%. For Patient 4, the available report documented an estimated ERBB2 copy number of 148.24. For Patients 1 and 2, the oncology medical records documented ERBB2 amplification by NGS, but the original complete molecular reports and additional specimen- and assay-level information could not be retrieved. Laboratory-specific numerical thresholds used to define amplification were unavailable for all four patients, and no missing values were estimated or imputed. HER2 immunohistochemistry and in situ hybridization results were unavailable for all four patients.

Best radiological response was retrospectively abstracted from contemporaneous radiology reports and oncology medical records and was reported using the terminology documented during routine clinical care. No blinded independent central radiological review was performed. The original post-treatment radiological source images were unavailable for independent re-review during revision. Progression-free survival was calculated from the first administration of T-DXd to documented radiological or clinical progression or death from any cause, whichever occurred first. Patients without documented progression were censored at the date of the last available disease assessment.

Treatment-related adverse events were retrospectively abstracted from oncology medical records and laboratory results and graded according to Common Terminology Criteria for Adverse Events version 5.0 when sufficient documentation was available. The absence of a documented event cannot exclude an event that was not recorded. Owing to the descriptive nature and small sample size of the series, no inferential statistical analysis was performed. The study was conducted in accordance with the Declaration of Helsinki. According to applicable local institutional policies, formal Ethics Committee approval was not required because the study involved retrospective analysis of anonymized clinical data collected during routine care, without additional research-related procedures. Written informed consent for publication was obtained from all patients.

## 3. Case Presentations

Patient 1 was a 65-year-old man with hypertension and metastatic urothelial carcinoma involving the lungs and lymph nodes. He received first-line cisplatin plus gemcitabine followed by avelumab maintenance. Following disease progression, T-DXd was initiated as second-line treatment at 5.4 mg/kg intravenously every three weeks. A computed tomography scan after four cycles documented a partial radiological response of the pulmonary metastases. After eight cycles, a subsequent assessment documented disease progression with new liver metastases, and T-DXd was discontinued. Progression-free survival was 8 months. The patient died in January 2025. Documented treatment-related adverse events included grade 3 leukopenia and fatigue. No interstitial lung disease or pneumonitis was documented.

Patient 2 was a 69-year-old man with hypertension and diabetes and metastatic urothelial carcinoma involving the lungs, liver, and lymph nodes. He had previously received pembrolizumab plus enfortumab vedotin, followed by cisplatin plus gemcitabine and erdafitinib. Following further disease progression, T-DXd was initiated as fourth-line treatment at 5.4 mg/kg intravenously every three weeks. A computed tomography scan after four cycles documented stable disease. After eight cycles, brain metastases were detected, indicating disease progression, and T-DXd was discontinued. Progression-free survival was 6 months. The patient died in February 2025. Documented treatment-related adverse events included grade 2 anemia, grade 2 diarrhea, grade 1 rash, and fatigue. No interstitial lung disease or pneumonitis was documented.

Patient 3 was a 66-year-old man with essential hypertension and a heavy smoking history who had been diagnosed with high-grade papillary urothelial carcinoma of the bladder in 2018. At T-DXd initiation, metastatic disease involved the liver. Molecular profiling of archival bladder tumor material identified ERBB2 amplification, with an estimated copy number of 10.4, together with a pathogenic ERBB2 p.Asp277Tyr (c.829G>T) variant with a variant allele frequency of 81.9%. Previous treatment consisted of platinum plus gemcitabine followed by avelumab maintenance, enfortumab vedotin, vinflunine, and paclitaxel. T-DXd was initiated as fifth-line treatment on 23 August 2024 at 5.4 mg/kg intravenously every three weeks. Stable disease was documented at radiological assessments performed approximately 4, 8, and 12 months after treatment initiation. At the last available treatment documentation, the patient had received 21 cycles. At the last verified clinical follow-up, more than 17 months after T-DXd initiation, he was alive. He was subsequently lost to follow-up; therefore, later treatment status, disease status, and survival status were unavailable. Progression-free survival was censored at the last documented disease assessment. The only clinically relevant documented treatment-related adverse event was grade 2 fatigue. No interstitial lung disease or pneumonitis was documented.

Patient 4 was a 76-year-old man with treated hypertension and papillary and solid urothelial carcinoma of the bladder with squamous differentiation, initially staged as pT4a G3 pN0. The diagnosis was established in July 2021, and metastatic lung involvement was documented on 18 March 2022. Molecular profiling of archival bladder tumor material identified marked ERBB2 amplification by NGS, with an estimated copy number of 148.24.

**Figure 1:**
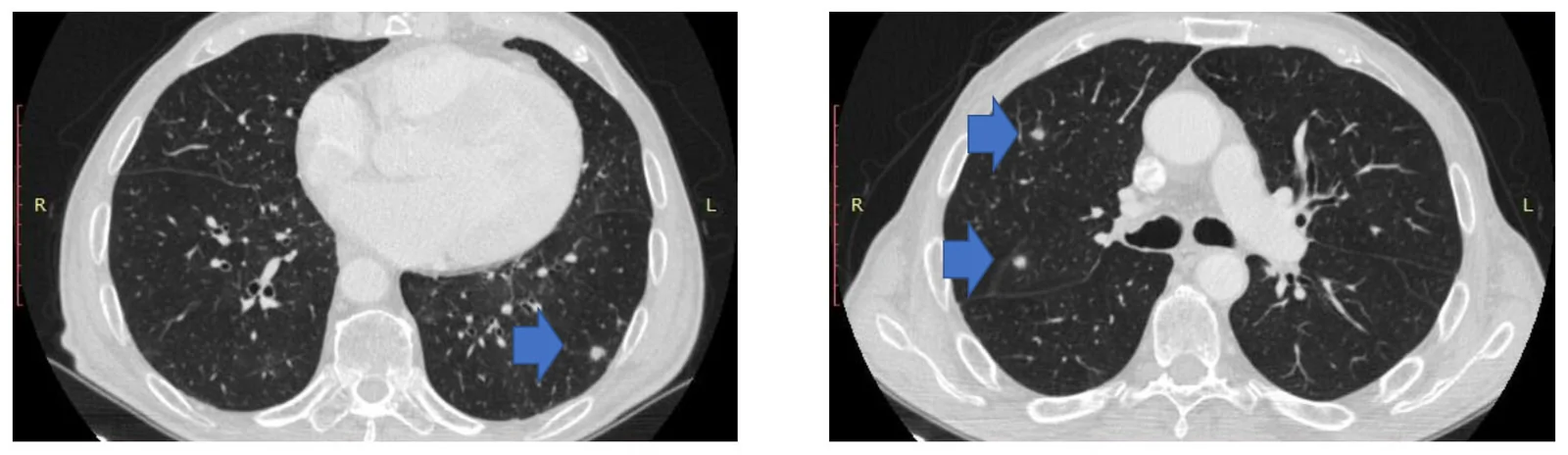
Representative baseline chest computed tomography images of Patient 4 before initiation of trastuzumab deruxtecan. Arrows indicate pulmonary metastatic nodules.

The patient received first-line platinum plus gemcitabine followed by avelumab maintenance, achieving a complete response. After subsequent progression, he received vinflunine, enfortumab vedotin, and paclitaxel, all discontinued because of progressive disease. T-DXd was initiated as fifth-line treatment on 12 April 2024 at 5.4 mg/kg intravenously every three weeks. A complete radiological response was documented in the contemporaneous medical record after four treatment cycles. Representative baseline chest computed tomography images are shown in Figure 1 and are provided for baseline illustration only. The patient received 12 cycles of T-DXd, with a progression-free survival of 8 months. T-DXd was discontinued after subsequent disease progression. The patient died in March 2025, approximately 11 months after T-DXd initiation. No clinically relevant treatment-related adverse events were documented, and no interstitial lung disease or pneumonitis was recorded.

Table 1 reports baseline demographic, clinical, and molecular characteristics of the four patients, while Table 2 reports treatment history, clinical outcomes, and treatment-related adverse events.

## 4. Discussion

This four-patient retrospective case series provides descriptive, patient-level information on T-DXd treatment in metastatic urothelial carcinoma selected by NGS-detected ERBB2 amplification. One patient received T-DXd in the second line, and three received it in the fourth or fifth line. Medical-chart review documented one complete response, one partial response, and two cases of stable disease. These observations cannot provide a reliable estimate of efficacy or safety and should not be interpreted as validating ERBB2 amplification as a predictive biomarker.

The cases should be interpreted within a treatment landscape that has changed rapidly. Enfortumab vedotin plus pembrolizumab is now the preferred first-line regimen for patients eligible for combination therapy [1,2]. Platinum-based chemotherapy remains relevant when this combination is unsuitable or unavailable, with avelumab maintenance for patients without progression after four to six cycles [1,3]. Because the patients in this series were treated during a period of therapeutic transition and had heterogeneous previous treatment sequences, their courses should not be read as representing a single contemporary standard sequence.

DESTINY-PanTumor02 has already established prospective activity of T-DXd in HER2-expressing solid tumors [6]. Its final bladder cancer analysis included 41 patients selected by HER2 immunohistochemistry, of whom 17 had an investigator-assessed objective response [7]. The contribution of the present series is therefore not a new efficacy signal. It is the description of treatment selection based on NGS-detected ERBB2 amplification when HER2 immunohistochemistry and in situ hybridization were unavailable, together with quantitative copy-number estimates in two patients, a co-occurring pathogenic ERBB2 variant in one patient, and previous enfortumab vedotin exposure in three patients.

The prevalence and interpretation of ERBB2 amplification in advanced urothelial carcinoma remain assay-dependent. A standardized analysis of 2024 advanced urothelial carcinoma specimens identified amplification in 11.6%, with the frequency increasing across HER2 immunohistochemistry categories but with amplification also present in some IHC 0 or 1+ tumors [4]. Earlier reviews reported substantial variation across studies [5]. These findings reinforce that genomic amplification and HER2 protein expression are related but non-interchangeable. The HERALD basket trial demonstrated activity of T-DXd in solid tumors selected by plasma cell-free DNA-detected HER2 amplification [8], but it did not establish a urothelial-carcinoma-specific genomic threshold. Patient 3 also harbored a pathogenic ERBB2 sequence variant; the relative contribution of amplification and mutation to his clinical course cannot be separated [9].

The activity of an antibody–drug conjugate depends on more than the target antigen. Antibody properties, internalization, linker stability, drug-to-antibody ratio, payload class, membrane permeability, and bystander activity can all affect efficacy and toxicity. T-DXd delivers a topoisomerase I inhibitor payload, whereas disitamab vedotin is a distinct HER2-directed ADC carrying monomethyl auristatin E. Disitamab vedotin has shown activity as monotherapy and in combination with toripalimab, including randomized first-line evidence [10,11,12]. Numerically different response rates across these studies should not be interpreted as direct comparative efficacy because the populations, HER2 definitions, treatment lines, and response-assessment methods differ.

The urothelial carcinoma ADC landscape also extends beyond HER2. Nectin-4-directed agents include enfortumab vedotin and the investigational agent bulumtatug fuvedotin, which produced a 54.1% objective response rate in the urothelial carcinoma cohort of a first-in-human phase I/II study [13]. TROP2-directed development includes sacituzumab govitecan and sacituzumab tirumotecan. In the randomized TROPiCS-04 trial, sacituzumab govitecan did not significantly improve overall or progression-free survival over chemotherapy despite a higher response rate [14], while sacituzumab tirumotecan remains under investigation after reporting activity in a pretreated cohort [15]. Other investigational programs have targeted EGFR/HER3, SLITRK6, and additional surface antigens [16]. These data illustrate why early response rates should be interpreted cautiously and why cross-trial rankings of ADCs are not appropriate.

Three patients in this series received enfortumab vedotin before T-DXd, representing sequential use of ADCs with different targets and different payload classes. The individual disease control documented during T-DXd treatment shows that prior exposure to enfortumab vedotin did not preclude subsequent treatment with a HER2-directed ADC in these cases. It does not establish an optimal sequence or exclude cross-resistance. Future studies should distinguish among the same target with a different payload, a different target with the same payload class, a different target with a different payload class, and true rechallenge with a previously administered ADC.

ADCs are also moving into earlier treatment lines and combination strategies. Enfortumab vedotin plus pembrolizumab and disitamab vedotin plus toripalimab demonstrate the potential of ADC–immunotherapy combinations [2,12]. The DAD phase I study of enfortumab vedotin plus sacituzumab govitecan provided evidence that two ADCs can be combined in a selected pretreated population, although its small phase I design does not establish an earlier-line standard [17]. Bispecific and dual-payload ADC formats may eventually address heterogeneous antigen expression or selected mechanisms of resistance; proposed combinations of targets such as Nectin-4 and HER2 remain investigational [18].

The limitations of this report are substantial. It includes only four patients, has no comparator, and relies on retrospective medical-chart data. Original complete molecular reports were unavailable for two patients, quantitative copy-number estimates were available for only two, and assay-specific amplification thresholds and additional specimen-level information could not be retrieved. HER2 immunohistochemistry and in situ hybridization were unavailable. For Patients 3 and 4, testing was performed on archival bladder tumor material, although the exact sampling dates could not be retrieved; the specimen source and sampling date were unavailable for Patients 1 and 2. Radiological responses were abstracted from contemporaneous reports and medical records; the original post-treatment source images were unavailable for independent re-review, so the post-treatment panels were removed, and the retained baseline figure is illustrative only. Exact dates needed to calculate the interval from T-DXd initiation to death were unavailable for Patients 1 and 2. For Patient 4, the exact day of death was unavailable; therefore, the interval from T-DXd initiation to death is reported as approximately 11 months. Patient 3 was alive more than 17 months after T-DXd initiation but was subsequently lost to follow-up. Adverse events were collected retrospectively, and undocumented events cannot be excluded.

Prospective studies should integrate standardized HER2 immunohistochemistry, in situ hybridization, quantitative tissue and/or plasma NGS, and longitudinal outcome assessment. Such studies are needed to determine whether genomic ERBB2 amplification adds predictive information beyond protein expression and to define how HER2-directed ADCs should be sequenced with other ADCs and systemic treatments.

## 5. Conclusions

In this four-patient retrospective series, chart-documented radiological disease control was observed during T-DXd treatment in patients selected on the basis of NGS-detected ERBB2 amplification, including three patients previously treated with enfortumab vedotin. These observations are descriptive and hypothesis-generating. The small sample size, retrospective design, absence of a comparator, incomplete molecular characterization, lack of HER2 immunohistochemistry and in situ hybridization, and absence of independent post-treatment radiological re-review preclude conclusions regarding efficacy, safety, or the predictive value of ERBB2 amplification.

## Figures and Tables

**Table 1:** Baseline demographic, clinical, and molecular characteristics of the four patients.

Patient	Sex	Age, Years	Comorbidities	Metastatic Sites	ERBB2 Molecular Findings
1	Male	65	Hypertension	Lung; lymph nodes	ERBB2 amplification by NGS
2	Male	69	Hypertension; diabetes	Lung; liver; lymph nodes	ERBB2 amplification by NGS
3	Male	66	Essential hypertension; heavy smoking history	Liver	ERBB2 amplification by NGS (copy number 10.4); pathogenic ERBB2 p.Asp277Tyr variant
4	Male	76	Treated hypertension	Lung	ERBB2 amplification by NGS (copy number 148.24)

The reported biomarker status refers to ERBB2 alterations detected by NGS. HER2 protein expression by immunohistochemistry and gene status by in situ hybridization were unavailable. Abbreviations: ERBB2, Erb-B2 receptor tyrosine kinase 2; HER2, human epidermal growth factor receptor 2; NGS, next-generation sequencing.

**Table 2:** Treatment history, clinical outcomes, and treatment-related adverse events.

Patient	Previous Systemic Therapies Before T-DXd	T-DXd Line	Best Response	Clinical Course	Toxicity
1	Cisplatin–gemcitabine → avelumab	Second	Partial response documented after 4 cycles	Liver progression after 8 cycles; PFS 8 months; died January 2025	Grade 3 leukopenia; fatigue
2	Pembrolizumab + enfortumab vedotin → cisplatin–gemcitabine → erdafitinib	Fourth	Stable disease documented after 4 cycles	Brain metastases after 8 cycles; PFS 6 months; died February 2025	Grade 2 anemia; grade 2 diarrhea; grade 1 rash; fatigue
3	Platinum–gemcitabine → avelumab → enfortumab vedotin → vinflunine → paclitaxel	Fifth	Stable disease at last available assessment	Stable disease documented at 4-, 8-, and 12-month assessments; alive at last verified clinical follow-up > 17 months after T-DXd initiation; subsequently lost to follow-up; PFS censored at last disease assessment	Grade 2 fatigue
4	Platinum–gemcitabine → avelumab → vinflunine → enfortumab vedotin → paclitaxel	Fifth	Complete response documented after 4 cycles	Received 12 cycles; PFS 8 months; died March 2025 (approximately 11 months after T-DXd initiation)	No clinically relevant treatment-related adverse events

Best radiological response was abstracted from contemporaneous radiology reports and oncology medical records. Adverse events were graded according to CTCAE version 5.0 when sufficient documentation was available. Abbreviations: CTCAE, Common Terminology Criteria for Adverse Events; PFS, progression-free survival; T-DXd, trastuzumab deruxtecan.

## Data Availability

The de-identified clinical information underlying this case series is not publicly available because of patient confidentiality and institutional restrictions. Limited de-identified data may be available from the corresponding author upon reasonable request and subject to institutional approval. Original post-treatment radiological source images and the complete molecular reports for Patients 1 and 2 are not available.

## Abbreviations

ADC, antibody–drug conjugate; CTCAE, Common Terminology Criteria for Adverse Events; ERBB2, Erb-B2 receptor tyrosine kinase 2; HER2, human epidermal growth factor receptor 2; IHC, immunohistochemistry; NGS, next-generation sequencing; PFS, progression-free survival; T-DXd, trastuzumab deruxtecan.
